# Paucity of Health Data in Africa: An Obstacle to Digital Health Implementation and Evidence-Based Practice

**DOI:** 10.3389/phrs.2023.1605821

**Published:** 2023-08-29

**Authors:** Sulaiman Muhammad Musa, Usman Abubakar Haruna, Emery Manirambona, Gilbert Eshun, Dalhatu Muhammad Ahmad, David Adelekan Dada, Ahmed Adamu Gololo, Shuaibu Saidu Musa, Abdulafeez Katibi Abdulkadir, Don Eliseo Lucero-Prisno III

**Affiliations:** ^1^ Faculty of Pharmaceutical Sciences, Ahmadu Bello University, Zaria, Nigeria; ^2^ School of Medicine, Nazarbayev University, Nur-Sultan, Kazakhstan; ^3^ College of Medicine and Health Sciences, University of Rwanda, Kigali, Rwanda; ^4^ Seventh-Day Adventist Hospital, Agona-Asamang, Ghana; ^5^ Faculty of Pharmaceutical Sciences, Kaduna State University, Kaduna, Nigeria; ^6^ Faculty of Pharmaceutical Sciences, Chulalongkorn University, Bangkok, Thailand; ^7^ Department of Nursing Science, Ahmadu Bello University, Zaria, Nigeria; ^8^ Department of Global Health and Development, London School of Hygiene and Tropical Medicine, University of London, London, United Kingdom

**Keywords:** health data, digital health, evidence-based practice, health policy, Africa

## Abstract

**Background:** Among the numerous challenges that Africa faces in improving its healthcare systems, the paucity of health data stands out as paramount. This study aims to examine the challenges related to the paucity of health data in Africa and its impact on the implementation of digital health and evidence-based practice. The findings of the study reveal that health data availability in Africa is both limited and frequently of poor quality. Several factors contribute to this concerning situation, encompassing inadequate infrastructure, a shortage of resources, and cultural barriers. Furthermore, the available data, despite its limitations, is often underutilized due to a lack of capacity and expertise in data analysis and interpretation.

**Policy Options and Recommendations:** To improve healthcare delivery in Africa, we recommend implementing novel strategies for data collection. It’s important to recognize that effective information technology service is crucial for enhancing healthcare delivery, and a holistic approach is necessary to achieve this.

**Conclusion:** This brief presents information to help policymakers develop long-term solutions to Africa’s health data poverty. Taking action based on this evidence can assist in addressing the problem.

## Background

Africa faces a myriad of challenges in improving its healthcare systems [1]. As the second-largest continent with a total land area of approximately 11,724,000 square miles (30,365,000 km^2^) and the second most populous continent with an estimated population of over 1.4 billion inhabitants, Africa’s representation in global health data is dismal [2–4]. As clinical practice is geared toward evidence-based medicine, patient-centered care, and value-based approach, health data has become a critical tool for clinicians to make informed decisions [5].

Health data has been defined as personal data concerning health. This includes all data about the health status of a subject and reveals information relating to the subject’s past, current, or future physical or mental health status. It also includes information provided by healthcare professionals as well as information generated by patients; from illnesses monitored through mobile applications and smart devices to screening tests and nutritional data [6, 7]. Although many indices indicate that data is becoming the backbone of evidence-based practice and digitized healthcare systems, a huge gap in health data representation is appealing within the continent with the largest share [8, 9]. A study viewed the lack of health data as “health data poverty” which they defined as “the inability for individuals, groups, or populations to benefit from a discovery or innovation due to insufficient data that are adequately representative” [9]. When appropriately harnessed, quality health data has the potential to achieve personalized treatments based on patients’ peculiar conditions, more focused health research and development, and improved insights for strategic planning.

The impact of healthcare data availability in Africa cannot be underestimated. The complex influencing factors and their interactions that affect the health of patients could be understood when health data is readily available, which in turn helps healthcare practitioners obtain significant knowledge and unravel patterns about health conditions, including rare diseases and those peculiar to the continent [5]. For example, the UK Biobank, Public Health England’s 1,000 genome project, the United States’ “All of Us” project, and the European Network of Cancer Registries, among others, have made genetic, clinical, biological, and socio-demographic health data available to clinicians in other parts of the world, thereby providing them with a better understanding of risk association and genetic causes of diseases [10]. When medical research and clinical trials fail to highlight specific demographics, valid data contribute to offering solutions to such unresolved problems [9]. Thus, the use of data in the health sector is critical for making evidence-based decisions [11]. Significant efforts have been put in place to consolidate the healthcare system, leading to improved health management information systems [12]. Unfortunately, most African countries allocate funds to address public health issues without adequate data, making it impossible to plan, monitor, and evaluate interventions [13]. As a result, the lack of health data has undermined Africa’s quest to improve healthcare delivery and services [14]. This article aims to discuss the impact of paucity and the importance of harnessing health data in Africa to build a resilient health system on the continent.

## Evidence

### Health Data Availability and Its Impact on Healthcare Delivery

The lack of adequate and high-quality health data in Africa has impeded the understanding of health-related issues [15]. Several studies have shown that healthcare providers can make informed decisions about the most effective and efficient practices for delivering care to patients by synthesizing available data [16]. In this way, individualized care can be properly designed, adapted, and implemented, and appropriate patient-specific management can be developed to meet the needs of the patient [17, 18]. The availability of health data with algorithms developed from artificial intelligence (AI) and machine learning (ML) can help predict and determine the appropriate diagnosis and prognosis of health conditions, the proper drug prescription, the treatment alternatives, and even strategies to reduce the duration of hospital stays and prevent re-hospitalization and recurrence of diseases [17, 19]. For example, the usage of available health data resulted in a 40 percent reduction in the length of hospital stay and a 50 percent improvement in the effectiveness of treatment elsewhere [20]. Health data in form of patient registries is fundamental to monitoring and evaluating the outcome of clinical treatment and the performance of health technology [21]. These benefits can lead to improved health outcomes and the health status of African patients and the general population.

Africa faces communicable and non-communicable diseases (NCDs), with communicable diseases accounting for the majority of deaths, while NCDs are projected to be the major cause of morbidity and mortality by 2030 [22]. It is pertinent to note that despite the huge burden of diseases, the lack of comprehensive and large-scale health data prevents researchers from understanding patterns in antimicrobial use and resistance to inform clinical decision-making and develop novel therapeutic agents that could reduce the impact of diseases on the continent [15].

Health data helps in the identification of relevant information, opens up new research opportunities and strategies, and creates innovative and cost-effective drugs or interventions for diseases [23]. Moreover, during clinical trials, the response and reaction of different categories of patients to drugs can be predicted with available data, thereby ensuring that approaches to detect the safety and efficacy of drugs are enhanced, and proper comparisons between intervention options are made [17, 23, 24].

The absence of detailed and up-to-date health data has caused obstacles in the management and assessment of healthcare policies, plans, and strategies in Africa [15]. In addition, this data is necessary for the formulation of policies, budgets, and strategies at various levels. Further, through the analysis of collected data, better evaluations, reviews, and modifications of health programs and policies can be made, thereby allowing for more effective monitoring and evaluation [25].

### Barriers to Sourcing Health Data in Africa

The African Union (AU) estimates that government health spending in Africa accounts for only 1% of total health expenditures, despite accounting for 24% of the global disease burden [13]. Several factors contribute to this, but the lack of precise data makes it difficult for governments and corporate donors to channel their spending on healthcare services. Health data sourcing in Africa has been a challenge due to the lack of a central electronic database and fragmented manual data gathering [26]. As a consequence, this leads to disparities in the collection and capture of inaccurate data, which could lead to the underrepresentation of key segments of an entire population. Since these data are used to develop and validate digital health technologies, data-driven interventions will become detrimental and biased for a particular set of populations [9].

Maintaining patient trust is key to building an efficient healthcare ecosystem. The patient should be assured that their data is safe from phishing, malware attacks, and vulnerability [27]. Until data security is adequately addressed, data collection remains a big problem, and health data poverty will persist. Africa’s growing population presents an opportunity for big data analytics in many business sectors, with healthcare examining the use of big data to find solutions for difficult diseases. Big data analytics requires high-quality internet services for efficient functioning. However, erratic network services pose a threat in that regard, and health data collection becomes a challenge, leading to health data poverty [28].

### Lack of Health Infrastructure and Technology

In Africa, healthcare centers are understaffed and under-equipped with Information Technology (IT) gadgets, patients have limited access to healthcare services, and there are communication gaps between patients and healthcare workers [29, 30]. While the use of mobile phones is only a subset of IT, it is worth noting that due to their low cost, ability to be easily carried around, ease of operation, and the durability of their batteries, particularly in rural areas, where there is unstable power supply, they have enhanced their utilization and penetration in Africa. The developing world has a 79% smartphone penetration rate with 2.2 billion mobile phones as of 2010 [31, 32]. Similarly, 50% of individuals living in rural areas were estimated to have mobile phones in 2012 [33]. The availability and penetration of mobile phones have been explored to minimize health problems and improve healthcare delivery. For instance, a study in Malawi captured the improvements recorded in enhancing healthcare service delivery through real-time information sharing. The approach used Short Message Service (SMS) communication to double enrolment for tuberculosis and HIV treatment [29]. A mobile phone-based system study was conducted in Rwanda, where the pregnancy was monitored through communication to reduce the delay in health-seeking behavior by sending an SMS to healthcare workers alerting them to timely medical support [32]. These initiatives could be expanded to include NCDs and other chronic infections.

Smartphone applications have reduced unnecessary hospitalizations and emergencies, thereby reducing healthcare spending, by encouraging self-management of chronic illness [9]. They have also minimized drug stock-outs by assisting in the procurement, ordering, and monitoring of drugs [33]. Despite the widespread adoption and use of IT in healthcare, these interventions are still relatively small. Lack of access to sophisticated mobile phones, under-skilled healthcare workers, unstable electricity, and the inability to implement large-scale healthcare reforms are some of the problems it faces [34]. There is no doubt that the implementation of IT for aggregating health data in Africa will improve healthcare delivery.

## Policy Options and Recommendations

Health data is cardinal in enhancing healthcare service delivery everywhere in the world, and the lack of it or its uneven distribution poses a threat to this course. The need for the enhancement of healthcare delivery in Africa calls for novel strategies to enhance data collection. It is imperative to note that enhancing healthcare delivery requires a holistic approach, and the importance of implementing an effective information technology service cannot be overestimated. Thus, we propose the following solutions.

### Health Data Automation

Automation of health data can address the problem of fragmented manual data collection and enhance processes. Healthcare providers and pharmaceutical companies can unlock new insights to help them make better, and more precise judgments. Therefore, this empowers them to respond quickly to life-saving measures. To meet the ever-changing needs of the sector, several activities, including gathering complete patient data, recommending a patient’s unique course of therapy, and giving stakeholders access to clinical data for their evidence synthesis, can be automated [35]. A study conducted in South Africa revealed that several benefits can be derived from the automation of health data in the delivery of healthcare. These include improved information security and patient confidentiality, as well as a reduction in healthcare costs [30].

### Public-Private Partnerships

Healthcare delivery in developing countries is increasingly being delivered through public-private partnerships. Partnerships in some African countries have yielded results and improved data collection. For instance, Program for Appropriate Technology in Health (PATH), a non-profit global health organization, and the Bill and Melinda Gates Foundation partnered with Tanzania, Ethiopia, and Malawi to create a Data Use Partnership [13]. These partnerships provided government agencies and global non-profits with vital data needed to address public health challenges in the region by providing funding for national health information systems [13]. The Gates Foundation’s commitment of about $300 million to facilitate the success of various public health programs in Tanzania would have limited impact without the data obtained through Data Use Partnerships [13]. Thus, private-public partnerships can be leveraged to expand health information gathering and usage across the African continent.

### Invest in Infrastructure and Technology

Infrastructure and technology are critical for the collection and utilization of health data in Africa. Adequate infrastructure and technology can improve the quality and accuracy of data, which in turn can inform better decision-making in the health sector. However, there are significant barriers to achieving this in many parts of Africa. A report on the mobile economy in sub-Saharan Africa found that 44% of the adult population is not connected to mobile internet service [36]. This lack of connectivity is a major barrier to the collection and analysis of health data.

This can be done by identifying key infrastructure and providing an alternative to it. For example, the lack of sufficient electric power supply can be tackled by providing solar energy to power the internet service engine.

### Promote Capacity Building and Open Data Initiatives

To improve the collection and analysis of health data for better patient care, the skills, and capacity of healthcare professionals and data analysts need to be enhanced through training, workshops, and educational resources on topics such as data management, analysis and visualization, governance, machine learning, natural language processing, and predictive modeling. Implementing standards and guidelines for data collection and storage can ensure data quality and reliability.

Open data initiatives make health data such as patient records, clinical trial results, and population health statistics available without any restrictions to researchers, healthcare providers, policymakers, and the public, in a format that can be easily accessed and used. This can be achieved through the use of open data portals and APIs. In Africa, open data initiatives can bridge the gap in data availability and accessibility, leading to better health outcomes for all [3].

### Foster a Culture of Data-Driven Decision Making

Promoting a culture of data-driven decision-making in healthcare is important to ensure that health data is used to inform policy and practice. This is achievable using data-driven tools and techniques, developing data literacy, and implementing data governance and management frameworks. Moreover, it can lead to improved patient care and more effective and efficient healthcare systems.

## Conclusion

The study highlights the issue of limited availability and poor quality of health data in Africa and its impact on implementing digital health and evidence-based practice. The paucity of data is due to factors such as inadequate infrastructure, limited resources, and cultural barriers. The lack of capacity and expertise in data analysis and interpretation also hinders the effective utilization of the available data. Addressing this issue requires collective efforts from governments, non-governmental organizations, and the private sector to improve data collection and utilization to ensure digital health solutions are tailored toward the needs of the African region and can have a maximum impact on improving health outcomes.
